# Oocyte aneuploidy rates in river and swamp buffalo types (*Bubalus bubalis*) determined by Multi-color Fluorescence In Situ Hybridization (M-FISH)

**DOI:** 10.1038/s41598-022-12603-9

**Published:** 2022-05-19

**Authors:** Alfredo Pauciullo, Carmine Versace, Angela Perucatti, Giustino Gaspa, Ling-Yu Li, Chun-Yan Yang, Hai-Ying Zheng, Qinyou Liu, Jiang-Hua Shang

**Affiliations:** 1 Department of Agricultural, Forestry and Food Sciences, University of Turin, 10095 Grugliasco (TO), Italy; 2 Laboratory of Animal Cytogenetics and Genomics, National Research Council (CNR), ISPAAM, 80056 Portici (NA), Italy; 3 Key Laboratory of Buffalo Genetics, Breeding and Reproduction Technology, Guangxi Buffalo Research Institute, Chinese Academy of Agricultural Sciences, Nanning, 530001 China; 4 State Key Laboratory for Conservation and Utilization of Subtropical Agro-Bioresources, Guangxi University, Nanning, 530004 China

**Keywords:** Cytogenetics, Infertility

## Abstract

Aneuploidy is one of the main causes of fetal and embryonic mortality in mammals. Nonetheless, its incidence in domestic ruminants has been investigated little. Indeed, no incidence data have ever been reported for water buffalo. To establish the incidence of aneuploidy in this species, we analysed in vitro matured metaphase II (MII) oocytes with corresponding first polar bodies (I PB) of the river (2n = 50) and swamp (2n = 48) buffaloes. For the first time, six river type probes (corresponding to chromosomes 1–5 and heterosome X), were tested on swamp buffalo metaphases using Multicolor-Fluorescent In Situ Hybridization (M-FISH) before their use on oocytes MII metaphases. Of the 120 total Cumulus Oocyte Complexes (COCs, 60 for each buffalo type) subjected to in vitro maturation, 104 reached the MII stage and were analysed by M-FISH. Haploid chromosome arrangement and visible I PB were observed in 89 of the oocytes (45 in river and 44 in swamp type). In the river type, the analysis revealed one oocyte was disomic for the chromosome X (2.22%). In the swamp type, one oocyte was found to be nullisomic for chromosome X (2.27%); another was found to be nullisomic for chromosome 5 (2.27%). We also observed one oocyte affected by a premature separation of sister chromatids (PSSC) on the chromosome X (2.27%). In both buffalo types, no abnormalities were detected in other investigated chromosomes. Based on merged data, the overall aneuploidy rate for the species was 3.37%. Oocytes with unreduced chromosomes averaged 1.92% across the two types, with 1.96% in river and 1.88% in swamp. The interspecies comparison between these data and cattle and pig published data revealed substantial difference in both total aneuploidy and diploidy rates. Reducing the negative impact of the meiotic segregation errors on the fertility is key to more sustainable breeding, an efficient embryo transfer industry and ex-situ bio-conservation. In this respect, additional M-FISH studies are needed on oocytes of domestic species using larger sets of probes and/or applying next generation sequencing technologies.

## Introduction

Meat and milk production, along with its use for draught, make the domestic water buffalo (*Bubalus bubalis*) among the most important global farm animals. Currently, its breeding is under expansion as well. Not only does its higher protein and fat content relative to other ruminants make it particularly suitable for cheesemaking^1^, but also its low fat content (leanness) allows its meat to be considered healthier than beef, although exceptions exist^2^. Cytogenetically, two types of domestic buffalo are differentiated based on their number of chromosomes (river type: 2n = 50 and swamp type: 2n = 48)^3^. Body morphology and behaviour also differ with this variation. Geographically, river type, bred for milk and meat production, is found in areas of India, Southwest Asia, Latin America, and the Mediterranean, while swamp type is established in Southeast Asia and China, where it is used mainly for draught and meat production.

Reproduction is a significant factor in determining livestock productivity. Reproductive health, known as fertility, is a complex quantitative trait that is usually assessed indirectly by different phenotypes (number of calvings, inter-calving intervals, conception rate, etc.). Generally, it is difficult to improve because of the low heritability of the trait and typically less than 5% in cattle^4–6^. One often insufficiently-considered genetic factor, known to cause nearly 70% of embryonic mortality in mammals, is the level of chromosomal abnormalities in germ cells^7^. In particular, aneuploidies -deviations from a normal chromosome number in a cell due to non-disjunction events- have been associated with infertility, miscarriage, perinatal mortality, and mental retardation in humans^8–10^, and with embryonic and foetal mortality in farm animals^7^.

The incidence of aneuploidy in the oocytes of domestic animals is limited. In the case of domestic buffalo, no information, as opposed to the most-often studied farm species of cattle^11–15^, pig^16–20^ or horse^21^. For these species, aneuploidy incidence has been established using both the conventional air-drying method^14,17^ and the more advanced molecular cytogenetic methods, including the Comparative Genomic Hybridization^16^, the Fluorescent In Situ Hybridization^12,13,20^ and the Whole Genome Sequencing^21^. Consideration of the growing international interest in water buffalo regarding the negative impact of chromosomal abnormalities on fertility, caused us to study and determine the frequency of aneuploidy in the oocytes matured in vitro of the two types of buffaloes (river and swamp) by a multi-colour FISH (M-FISH).

## Results

**Figure 1:**
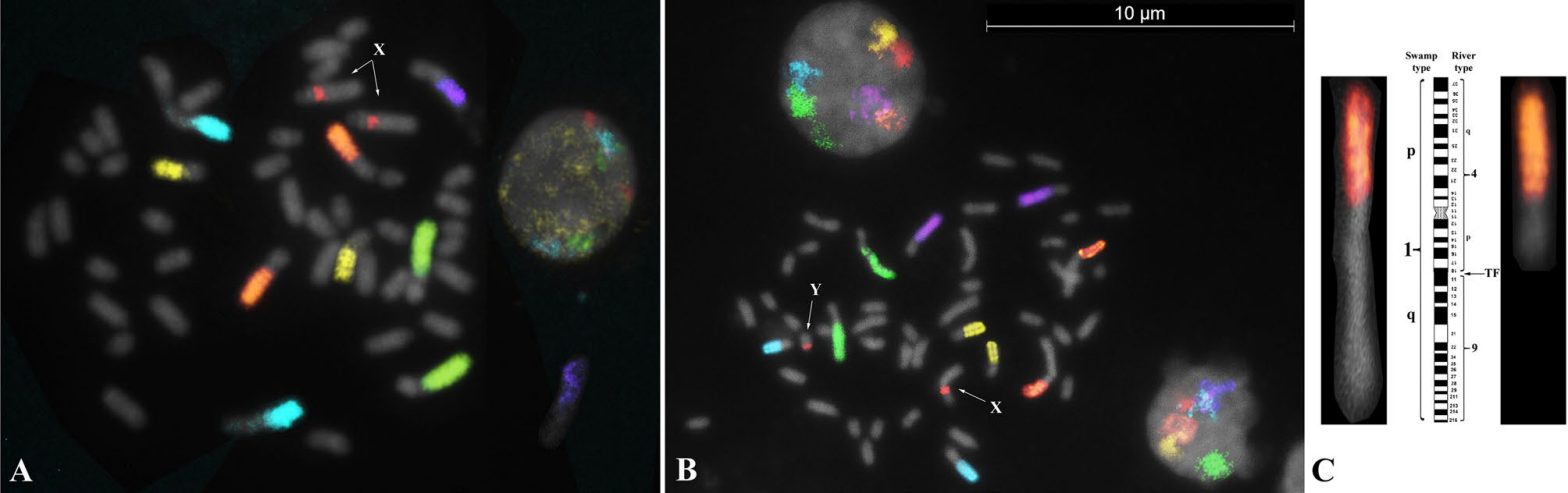
Multicolour hybridization with 6 DNA probes on normal buffalo metaphase spreads, (**A**) river type (*Bubalus bubalis*, 2n = 50,XX); (**B**) swamp type (*Bubalus bubalis*, 2n = 48,XY). The X probes showed a signal also on the Y-chromosome; (**C**) details on straightened swamp chromosome 1 (left) generated by the tandem fusion (TF) of the chromosomes 4 (right, upside down) and 9 of the river type. In the middle the R-banded ideogram of the chromosome.

Chromosome specific painting probes were validated on both river (2n = 50,XX) and swamp buffalo (2n = 48,XY) metaphase spreads. Both individual (Supplementary Fig. 1) and sequential hybridisations (Fig. 1) produced strong signals on all investigated chromosomes, so we used them for the oocytes analysis.

**Figure 2:**
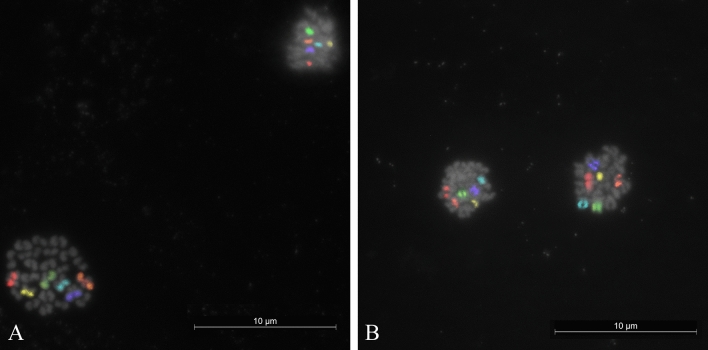
Multi-colour FISH showing the six signals in both metaphases and corresponding first polar bodies of in vitro-matured secondary oocytes as indication of a normal chromosomal segregation in (**A**) river buffalo (MII,25,X); (**B**) swamp buffalo (MII,24,X).

**Figure 3:**
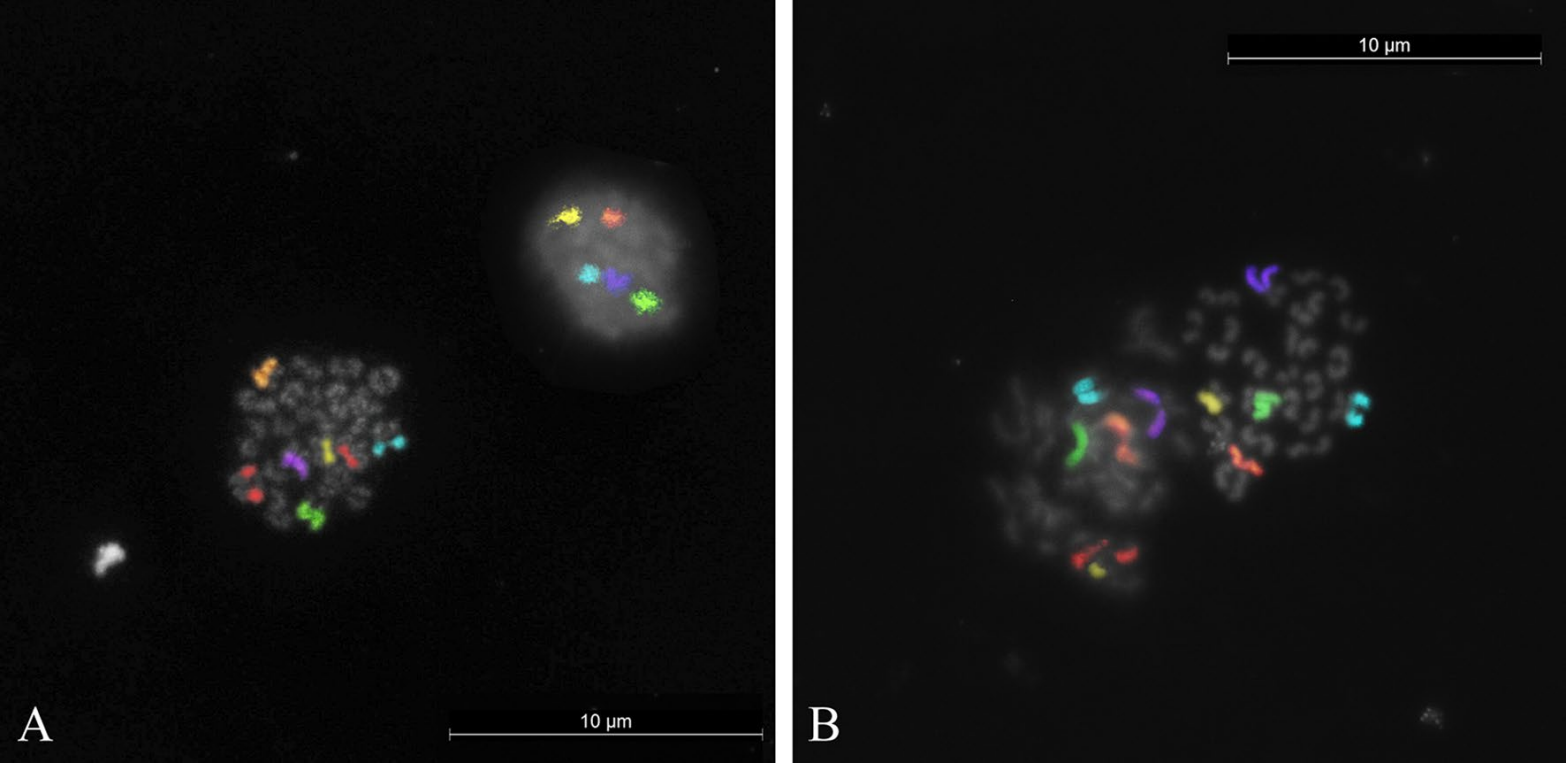
Aberrant secondary oocyte metaphases for the heterosome X (red color); (**A**) disomy in river buffalo (MII,25,X,+X); (**B**) nullisomy in swamp buffalo (MII,24,-X).

**Figure 4:**
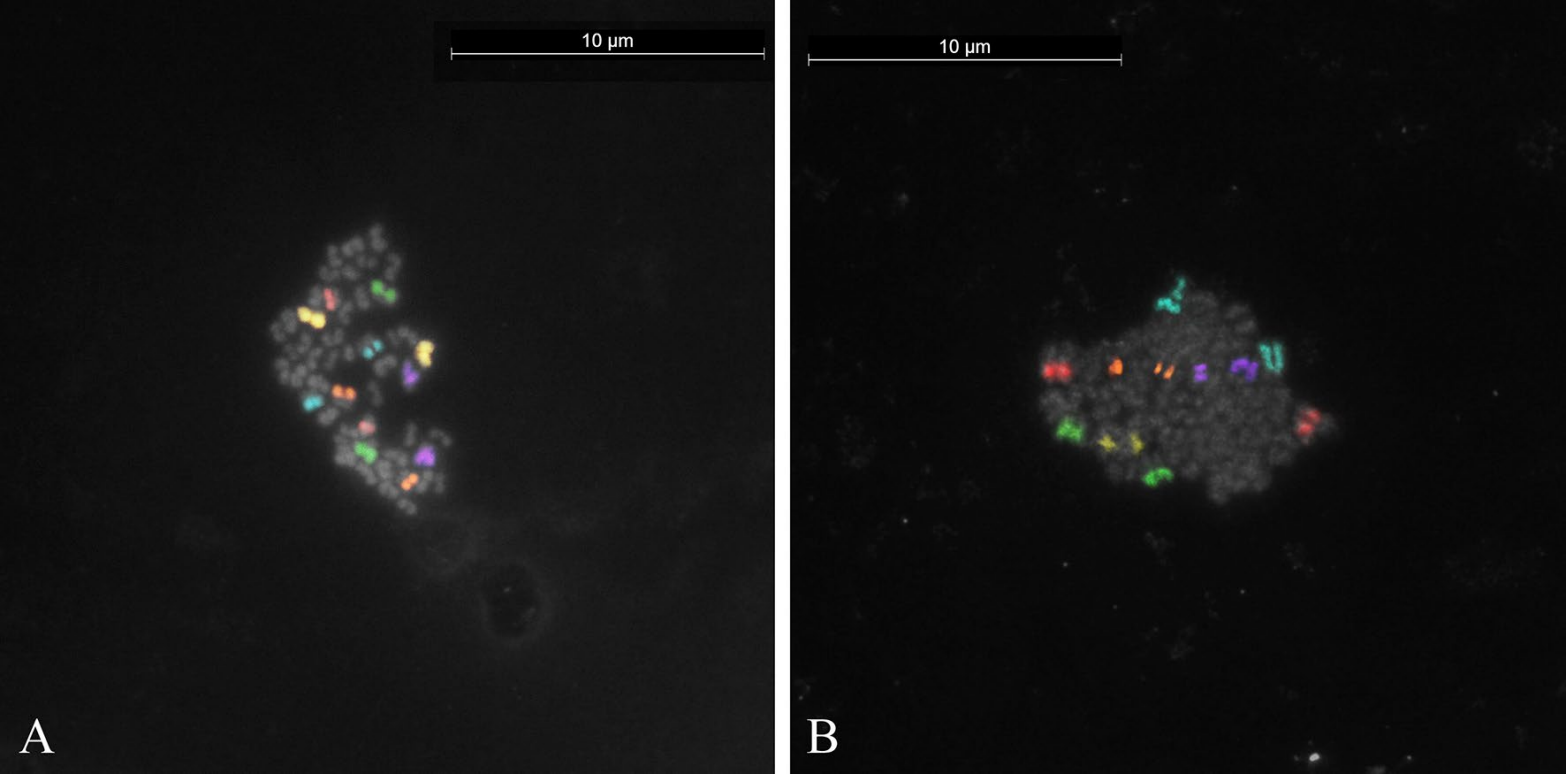
Unreduced secondary oocyte metaphases in (**A**) river buffalo (MII,50,XX); (**B**) swamp buffalo (MII,48,XX).

**Figure 5:**
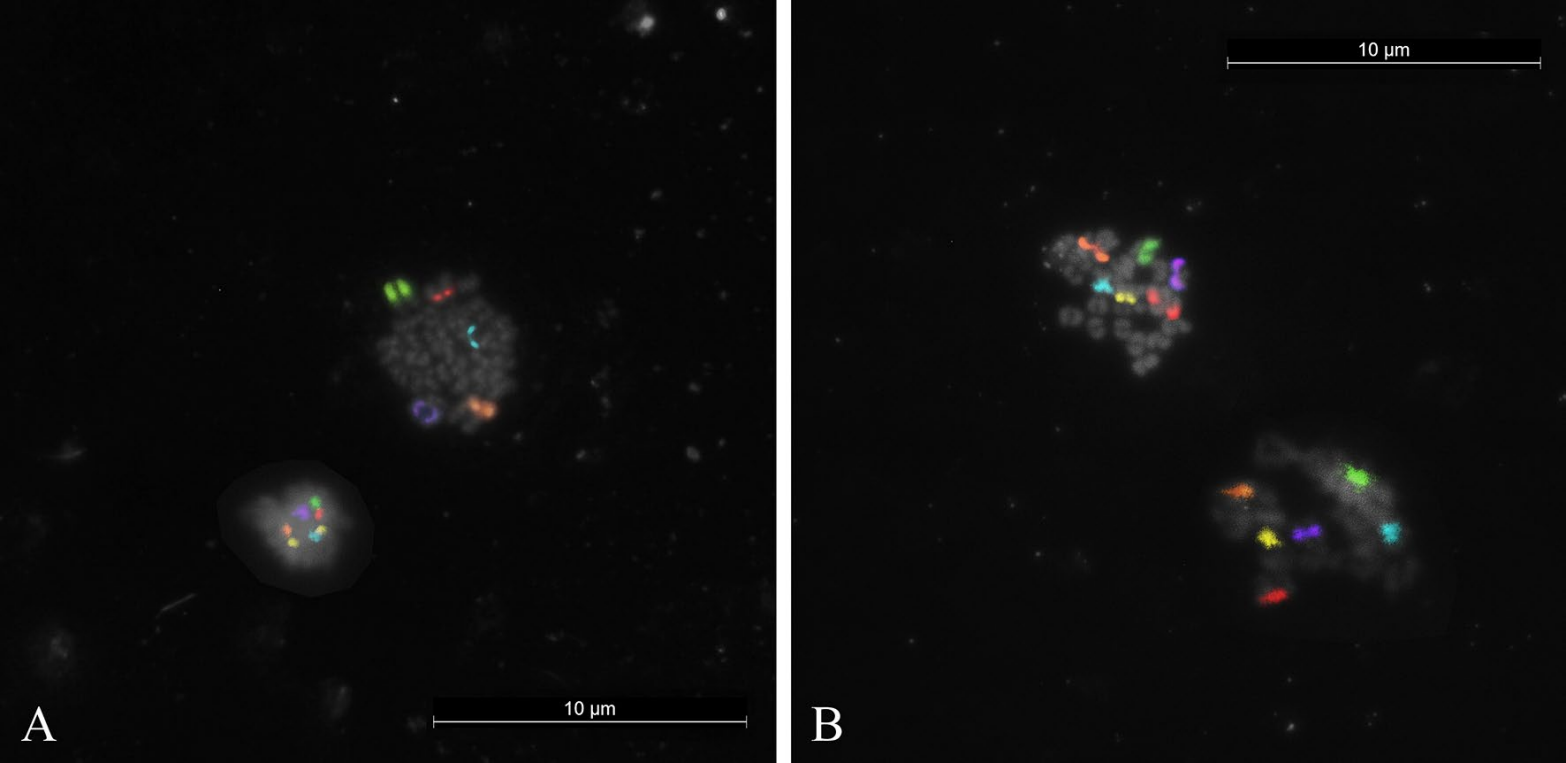
Metaphases and corresponding first polar bodies of in vitro-matured secondary oocytes of swamp buffalo after multicolour FISH showing (**A**) nullisomy for chromosome 5 (MII,24,X,-5) (yellow); (**B**) PSSC for chromosome X (MII,24,X,−X,+Xcht,+Xcht) (red).

Results are reported in the Table 1 and in the Figs. 2, 3, 4, 5. Out of 120 cumulus oocyte complexes—COCs—(60 for each buffalo type) used for in vitro maturation (IVM), 104 reached the MII stage (51 river and 53 swamp). The efficiency of the IVM procedure averaged > 80% in the both buffalo types (85.0% river and 88.3% swamp).

**Table 1:** River and swamp buffalo secondary oocytes matured in vitro and analysed by sequential M-FISH with 6 chromosome specific painting probes for aneuploidy detection.

Type	Breed	Number of analysed oocytes									
		Selected for IVM	Total MII analysed (a)	Unreduced (% on a)	Reduced			Aneuploid			PSSC (% on c)
					Total (b) (% on a)	− PB (% on b)	+ PB (c) (% on b)	Nullisomic (% on c)	Disomic (% on c)	Total (% on c)	
River	Murrah	60	51	1 (1.96)	50 (98.03)	5 (10.00)	45 (90.00)	–	1^x^	1	–
Swamp	Local	60	53	1 (1.88)	52 (98.11)	8 (15.38)	44 (84.61)	1^x^ + 1^5^	–	2	1^x^
	Total	120	104	2 (1.92)	102 (98.07)	13 (12.74)	89 (87.25)	2 (2.25)	1 (1.12)	3 (3.37)	1 (1.12)

*IVM* in vitro maturation, *PB* polar body, *PSSC* premature separation of sister chromatids.

(a) Number of matured oocytes;

(b) Number of oocytes with reduced chromosome number;

(c) Number of oocytes with reduced chromosome number and analysable PB.

For the river buffalo, among the 51 oocytes at the MII stage, 50 showed haploid chromosome arrangements (Supplementary Fig. 2). However, the chromatin of the PB in five of these oocytes was not found, so they were excluded from the analysis. FISH was achieved on the 45 oocytes that showed the corresponding the first PB (Fig. 2A). One oocyte (2.22%, 1/45) was found to be disomic for the chromosome X (Fig. 3A), whereas no abnormalities were observed for any other investigated chromosomes. Overall, the frequency of aneuploidy (nullisomy and disomy) corresponded to the disomy value (2.22%). In addition, one secondary oocyte (1.96%, 1/51) exhibited an unreduced, diploid, set of chromosomes (Fig. 4A).

For the swamp type, 52 of the 53 oocytes that reached MII stage displayed the haploid chromosome set, but the first PB was not found in eight of these, so FISH analysis was performed on only 44 MII with the corresponding first PB (Fig. 2B). Nullisomy for chromosome X and 5 was found in two oocytes (4.54%, 2/44) exhibited in Figs. 3B and 5A, respectively. No other abnormalities were observed for the other chromosomes. Therefore, the incidence of aneuploidy corresponded to that of nullisomy (4.54%). In addition, one haploid oocyte (2.27%, 1/44) was affected by a premature separation of sister chromatids (PSSC) of chromosome X (Fig. 5B). The other secondary oocyte out of the starting 53 (1.88%) was found with an unreduced chromosome number (Fig. 4B).

Merging the data for the two buffalo types, we found that two oocytes out of 104 (1.92%) were unreduced; whereas out of 89 MII analyzed, two oocytes were nullisomic (2.25%), and one oocyte was disomic (1.12%), which resulted as a 3.37% overall aneuploidy rate for the species.

## Discussion

This study is the first report of aneuploidy rates in the secondary oocytes of two types of water buffalo (river and swamp) obtained using six chromosome-specific painting probes in two sequential rounds of hybridisation. It is also the first reported study using M-FISH for the analysis of aneuploidies in oocytes. It was prompted following a previous study in which we explored this approach in cattle^22^. That study focused on the cross-hybridisation of a larger set of probes on lymphocyte metaphases in species including the river buffalo. However, only a few oocytes were used to test the method on female cattle germ cells. Despite the production of limited data, the M-FISH probes provided evidence of maturation differences between MII oocytes (correct chromosomal segregation and visible first PB) and MI oocytes (occurrence of tetrads and absence of the corresponding first PB). The results aroused our interest in buffaloes and encouraged us to investigate the meiotic chromosomal abnormalities in both river and swamp types.

Exploration of the water buffalo required that we first test the six probes on lymphocytes metaphases. For the river type, we confirmed the hybridisations reported in the first study^22^ (Fig. 1A), whereas for the swamp type, the probes were tested for the first time and produced satisfactory results (Fig. 1B). The main difference between the two buffalo types lies into swamp chromosome 1. Specifically, a telomere-centromere tandem fusion occurred between two chromosomes identified as 4p and 9 in the river buffalo karyotype (2n = 50), which reduced diploid number (2n = 48) in swamp buffalo^3^. The much longer swamp chromosome 1, relative to all other sub-metacentric chromosomes of the river type, allowed the probe used as 4q in the river buffalo (Table 2) to identify the short arm of chromosome 1 in the swamp type (Fig. 1C). The X probes also produced a signal on the Y-chromosome in the 1.8–1.10 region, indicating the presence of X-specific DNA sequences recognised by the probe (Fig. 1B). This finding had already been reported in the river buffalo^23^, now our data confirmed the same hybridisation existed in the swamp type.

**Table 2:**
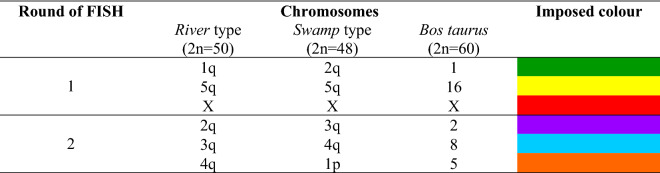
Chromosome specific painting probes used in the sequential M-FISH for the river and swamp buffalo. Super imposed colours and homologous chromosomes in *Bos taurus*.

Our result highlights the importance of FISH as a method for determining structural rearrangements at the level of the chromosomal band that are difficult to detect by classical karyotyping. Notwithstanding, FISH is important for several other reasons: understanding the nature of chromosomal reorganisation in evolution^24^, removing ambiguity in chromosome identification like micro-chromosomes^25^, supporting bioinformatics as complementary approach in genome assembly^26^, and providing information on germ cells aneuploidies^27–30^, etc. In fact, since the 2000s, chromosome-specific painting probes in M-FISH screening has informed human pre-conception^31^ and pre-implantation genetic testing^32^. The tool reduces miscarriages and aneuploidy births, and increases implantation and pregnancy rates.

The assessment of aneuploidy rates in domestic farm animals must similarly be considered as an indispensable step. It is critical if a more sustainable production of embryos for the embryo transfer industry and if reproductive health trends, management errors and environmental hazards in selective trait breeding are to be monitored. Given this perspective, the probes used in this study gave strong hybridisation signal both in MII and the first PB of secondary oocytes (Figs. 2, 3, 4, 5). The total aneuploidy rates for the six chromosomes tested were 2.22% in river and 4.54% in swamp buffalo (Table 1). Despite the double frequency in the swamp type, the difference was not significant (p = 0.98). However, it is interesting to notice that the disomy was the only aberration for determining the aneuploidy rate in river type and conversely, nullisomy was the only abnormality to affect the total rate in swamp buffalo. After combining the data, the overall aneuploidy rate across both buffalo types equalled 3.37% (Table 1), which is above that observed in four investigated cattle breeds (2.25%)^12,13^, but still not significant (p = 0.81). Also when the comparison was limited only to chromosome X, analysed with the same FISH method in both species, the difference in the incidence of aneuploidy (2.25% vs 0.5% in buffalo and cattle, respectively) was non-significant (p = 0.32).

Analysing the frequency of aneuploidy among chromosomes, the first five couples of submetacentric chromosomes were almost never involved in non-disjunction events, whereas the heterosome X exhibited twice the incidence compared to submetacentric chromosome 5 (2.25% vs 1.12%, respectively). Although not statistically significant, the difference indicates that interchromosomal differences exist in the frequency of nondisjunction, as observed in other species. For instance in cattle, the chromosome X aneuploidy rate of 0.5% was much lower that 1.75% non-disjunction frequency in chromosome 5^13^, which was not aneuploid in this study because it corresponded to the buffalo chromosome 4q (Table 2). In pig, at least three studies on secondary oocytes demonstrated an unequal involvement in non-disjunction events, with a prevalence in smaller chromosome pairs^19^ and in chromosome 10^17,33^. In humans, similar results were found. Various analytical methods (Classical cytogenetics, FISH, CGH) all demonstrated that even though all chromosomes can participate in aneuploidy events, the smaller ones seem to be preferentially affected during both meiotic divisions^34^.

No small chromosomes were investigated in the present study, so we have no evidences in buffalo. Indeed, in this species we must consider that each chromosome might have the same probability for meiotic errors. Under this assumption, the average aneuploidy rate can be established as 0.56% per chromosome (3.37% per six chromosomes). Following the logic means that nearly 14% (0.56% × 25 or 24 haploid chromosomes according to the buffalo type) of the oocytes matured in vitro might give way to meiotic errors. While this value is almost three-fold the average cattle aneuploidy rate (5.2%) assessed by conventional cytogenetic methods^11,15,35^, it is nearly half the rate assessed using FISH (30%, 1% per chromosome). In both cases, the rates were established in intensively selected breeds^12^ and in local breeds^13^. Total aneuploidy values reported in pig oocytes matured in vitro and analysed by FISH average about 27% (average rate of 1.4% per chromosome)^20^, while conventional methods have produced an average frequency between 4 and 5%^18,19^. In other mammalian species, the rates of aneuploidy assessed by conventional methods were 5.8% in the horse^7^ and rabbits^36^, 1.8% in hamster^37^, and 2.7% in mouse^38^.

Consideration of the data of the present study and those reported in other species suggests that the FISH method overestimates aneuploidy rates when compared with the classical cytogenetic approaches. However, it would be premature to come to such a conclusion for two reasons. The first is that the data were extrapolated from only a single analysis of a few chromosomes and the second reason is that it assumes that no inter-chromosomal differences exist with the rate of non-disjunction. Further studies are necessary in all farm animals to cover a major fraction of their genomes by a higher number of chromosome-specific FISH probes.

Limited domestic animal studies prohibit further comparisons. Nonetheless, despite the meagre literature analysing oocytes by FISH, we can state that the method is extremely accurate. In humans, the non-disjunctions rate is highly variable with peaks up to 47%^29^, although recent high resolution Next Generation Sequencing (NGS) has more precisely estimated it at about 19.7%^39^, or an average rate per chromosome of 0.85%, a similar value to that estimated in the present study by FISH.

The incidence of diploidy was 1.96% in river *vs* 1.88% in swamp buffalo (Table 1). On average, the frequency of diploidy in buffalo oocytes (1.92%) is not significantly different (p = 0.149952) from the level of diploidy evidenced in Egyptian buffalo (6.50%) by conventional Giemsa staining^40^, but it is significantly (p = 0.001022) lower than the diploidy rate (10–12%) reported in cattle oocytes analysed by FISH^13^ and conventional Giemsa staining^14,15^. The level of buffalo unreduced oocytes is also significantly (p < 0.00001) different from the level of diploidy (25–29%) detected in pig oocytes analysed by FISH^20^ and by classical methods^17^. In general, diploidy has been considered the most frequent abnormality reported in oocytes matured in vitro^7,11,15,17,20,35^. Relative to other species, the number of secondary abnormalities analysed by FISH in buffalo is low (89 in buffalo, 400 in cattle, 1349 in pig).

The low diploidy and aneuploidy rates per chromosome found in buffalo oocytes deserves attention. Chinese buffaloes (both river and swamp type) are not subjected to worldwide selection programs done for most cattle and pig breeds. Furthermore, buffaloes still keep their rustic nature in the reproductive behaviour^41^, whereas the breeding seasonality of cattle has been greatly influenced by intensive selection that, combined with environmental and management factors, caused fertility reduction, as observed over decades in dairy breeds^6^. Indeed, maintained rusticity and limited (or absent) genetic improvement in a species may indirectly preserve the meiotic biological mechanisms (crossing-over, sister chromatid cohesion, spindle assembly/disassembly, etc.) that on alteration generate segregation errors and chromosomal aberrations^42^.

Premature separation of sister chromatids (PSSC), driven by cohesion weakening, is considered one of the most common segregation errors leading to aneuploidy^43^. A balanced PSSC indicates a tendency to the non-disjunction, whereas an unbalanced PSSC may lead to aneuploid embryos in 50% of the cases, depending on the behaviour of the extra chromatid during the second meiotic division. We found that the X chromosome in the swamp buffalo (incidence of 1.12%) had a balanced PSSC (Fig. 5B). This data is consistent with the total PSSC error rate in cattle (1.25%), although a slight difference was observed for the specific chromosome (0.25%)^13^. No unbalanced PSSC oocytes were found.

This study demonstrated that M-FISH is an effective method for the detection of chromosomal differences and abnormalities in little investigated species like the buffalo. The M-FISH allowed us to confirm two events that differentiate the swamp from the river type of buffalo -the chromosomal fusion that occurred during the karyological evolution and the presence of X-specific DNA sequences on the Y-chromosome. Additionally, the analysis of oocytes aneuploidies indicated other differences on the examined individual chromosomes and on the type of occurring aneuploidy. The interspecific comparison between our data and previous studies on cattle and pigs revealed substantial differences both in total aneuploidy and diploidy rates. While the reported data narrowed a knowledge gap in the field of domestic animal reproductive cytogenetics, additional research on aneuploidy in oocytes is called for and should be conducted using larger sets of chromosomal probes by M-FISH or new sequencing technologies (NGS) as recently used for horse^21^. Application of NGS to the first polar bodies would extend segregation error information along the entire genome. Available preserved oocytes could be used for in vitro embryos production that contribute to a more sustainable embryo transfer industry and/or ex-situ biodiversity conservation programs that select endangered species gametes for cryopreservation.

## Methods

### Ethics declarations

Slaughtering procedures were conducted according to the rules of animal care of the People’s Republic of China. All experimental procedures were conducted following to the Animal Ethics Committee of the Guangxi Buffalo Research Institute. No further approval was required for this study because the probes were obtained from an existing collection and their production was already approved in 2014 by the Committee on the Ethics of Animal experiments of the CNR-ISPAAM (Permit Number: 0000391-18/03/2014).

### Collection of ovaries

Sampling was performed between September and October of 2019 in Guangxi Region (China). Forty ovaries from 20 female buffaloes (on average 36 ± 2 months old) were collected from a local slaughterhouse. Ten of the animals were of the Chinese Murrah breed (river type) and the other 10 females were from a local Chinese breed (swamp type). The ovaries were collected within 30 min after slaughter, stored in Phosphate Buffered Saline (PBS) supplemented with 0.05 mg/ml streptomycin and 0.06 mg/ml penicillin at 30–35 °C, and transported to the laboratory within 2 h. All the visible follicles were aspirated using a 17-gauge needle attached with a 10 ml syringe.

### In vitro maturation of cumulus oocyte complexes (COCs)

The COCs were in vitro matured according to the method described by Yang et al.^44^. Briefly, COCs were observed under stereoscopic microscopy (Nikon SMZ 1500). Only COCs with homogeneous cytoplasm, uniform texture and more than one compact layer of cumulus cells were chosen for the in vitro maturation (IVM). Every 10 COCs were transferred to each fresh droplets (40 µl) of maturation medium consisting of TCM-199 + 10% foetal bovine serum, supplemented with 5 µg/ml of follicle-stimulating hormone (FSH), 10 µg/ml luteinizing hormone (LH), 0.2 mM sodium pyruvate, 1 µg/ml estradiol (E2), 50 µM cysteine and 25 ng/ml epidermal growth factor (EGF). The droplets were covered with sterile mineral oil, and cultured under a humidified atmosphere of 5% CO_2_ at 38.5 °C for 22–24 h.

### Oocyte denuding and metaphases fixation

After maturation, the oocytes were incubated in a 1 mg/ml hyaluronidase solution for 3–5 min at 37 °C to remove the cumulus cells. Once denuded, the oocytes were washed in PBS and treated with two consecutive hypotonic solutions: sodium citrate at 0.8% (wt/vol) and KCl 0.56% (wt/vol), each for 3 min. Each oocyte was transferred individually at the centre of a pre-cleaned slide and immediately subjected to fixation with a cold methanol/glacial acetic acid (1:1) solution. By gently and slowly dropping the fixative solution on the slide, the *zona pellucida* of each oocyte was chemically opened, which then allowed the release of the metaphase and polar body (PB) that became fixed once air-dried. The slides were maintained at − 20 °C until analysis.

### Probe preparations and multicolour FISH

The species-specific chromosome painting probes used in this study belonged to the collection of Pauciullo et al.^22,45^. In total, six probes were used. Five corresponding to the q-arms of sub-metacentric autosomes (1q, 2q, 3q, 4q and 5q) of river type buffalo (2n = 50) plus the heterosome X that mapped region q21-25.

Lymphocyte cell cultures from four buffalo bulls (two river and two swamp) were prepared according to the standard cytogenetic techniques^46^. The buffaloes were karyotyped and were karyologically normal. Therefore, each probe was hybridised on metaphase plates for validation, first individually and then simultaneously with two sequential steps of FISH with three probes each. Only after this preliminary test the probes were used for oocyte analysis.

Each probe was labelled separately with Spectrum Orange-dUTP and Spectrum Green-dUTP (Abbott, USA) in a DOP-PCR reaction using 2 µl of the original probe template^22^. The labelling scheme is reported in the Table 2.

Two sequential rounds of hybridisation were performed on the same slide. The whole FISH procedure including probe precipitation, probe and slides denaturation, incubation and washing steps were accomplished according to Pauciullo et al.^22^.

### Fluorescence analysis and scoring

The slides were observed at 100 × magnification with a Nikon Eclipse E600 fluorescence microscope equipped with Fluorescein isothiocyanate (FITC), Texas Red (TXRD) and 4′,6-diamidino-2-phenylindole (DAPI) specific filters, assembled with a Leica DSC450 camera and controlled by the Leica LAS EZ software. Digital images were captured in grey-scale, and then the colours were superimposed for the final observation and assessment of the chromosomal arrangement.

To avoid possible bias, reduced secondary oocytes without the corresponding first polar bodies were excluded from the analysis. As an assessment criterion, an oocyte was defined as nullisomic when at least one hybridisation signal of the six chromosome specific probes (1q, 2q, 3q, 4q, 5q and X, taken as the reference river type) was absent from the MII plate, but present in duplicate in the corresponding first PB. In the same way, an oocyte was defined as disomic when at least one of the hybridisation signals was present twice in the MII, but absent in the PB. Double signals of all probes and the lack of PB was considered as a non-reduced chromosome arrangement. Chi-square analysis with Yates’ correction was used for the statistical analysis of data.

## Supplementary Information

Supplementary Figures.

## Data Availability

All data generated or analysed during this study are available from the corresponding author upon reasonable request.

## References

[1] Pauciullo A (2021). A novel duplex ACRS-PCR for composite CSN1S1–CSN3 genotype discrimination in domestic buffalo. Ital. J. Anim. Sci..

[2] Di Stasio L, Brugiapaglia A (2021). Current knowledge on river buffalo meat: A critical analysis. Animals.

[3] Di Berardino D, Iannuzzi L (1981). Chromosome banding homologies in Swamp and Murrah buffalo. J. Hered..

[4] Berglund B (2008). Genetic improvement of dairy cow reproductive performance. Reprod. Domest. Anim..

[5] Cammack K, Thomas M, Enns R (2009). Reproductive traits and their heritabilities in beef cattle. Prof. Anim. Sci..

[6] Walsh S, Williams E, Evans A (2011). A review of the causes of poor fertility in high milk producing dairy cows. Anim. Reprod. Sci..

[7] King WA (1990). Advances in Veterinary Science and Comparative Medicine.

[8] Hassold T, Hunt P (2001). To err (meiotically) is human: The genesis of human aneuploidy. Nat. Rev. Genet..

[9] Hecht F, Hecht BK (1987). Invited editorial comment on the article by Salamanca-Gómez et al.: Environmental chromosome damage. Am. J. Med. Genet..

[10] Martin RH, Ko E, Rademaker A (1991). Distribution of aneuploidy in human gametes: Comparison between human sperm and oocytes. Am. J. Med. Genet..

[11] Lechniak D, Świtoński M, Sosnowski M (1996). The incidence of bovine diploid oocytes matured in vitro. Theriogenology.

[12] Nicodemo D (2010). Frequency of aneuploidy in in vitro-matured MII oocytes and corresponding first polar bodies in two dairy cattle (*Bos taurus*) breeds as determined by dual-color fluorescent in situ hybridization. Theriogenology.

[13] Pauciullo A (2012). Similar rates of chromosomal aberrant secondary oocytes in two indigenous cattle (*Bos taurus*) breeds as determined by dual-color FISH. Theriogenology.

[14] Sosnowski J, Świtoński M, Lechniak D, Moliński K (1996). Cytogenetic evaluation of in vitro matured bovine oocytes collected from ovaries of individual donors. Theriogenology.

[15] Yadav B, King W, Xu K, Pollard J, Plante L (1991). Chromosome analysis of bovine oocytes cultured in vitro. Genet. Sel. Evol..

[16] Hornak M (2011). Frequency of aneuploidy related to age in porcine oocytes. PLoS One.

[17] Lechniak D (2007). Gilts and sows produce similar rate of diploid oocytes in vitro whereas the incidence of aneuploidy differs significantly. Theriogenology.

[18] McGaughey R, Polge C (1971). Cytogenetic analysis of pig oocytes matured in vitro. J. Exp. Zool..

[19] Sosnowski J, Waroczyk M, Switonski M (2003). Chromosome abnormalities in secondary pig oocytes matured in vitro. Theriogenology.

[20] Vozdova M (2001). Frequency of aneuploidy in pig oocytes matured in vitro and of the corresponding first polar bodies detected by fluorescent in situ hybridization. Theriogenology.

[21] Shilton CA (2020). Whole genome analysis reveals aneuploidies in early pregnancy loss in the horse. Sci. Rep..

[22] Pauciullo A (2014). Sequential cross-species chromosome painting among river buffalo, cattle, sheep and goat: A useful tool for chromosome abnormalities diagnosis within the family Bovidae. PLoS One.

[23] Di Berardino D (2004). Sexing river buffalo (*Bubalus*
*bubalis* L.), sheep (*Ovis*
*aries* L.), goat (*Capra*
*hircus* L.), and cattle spermatozoa by double color FISH using bovine (*Bos*
*taurus* L.) X-and Y-painting probes. Mol. Reprod. Dev. Incorp. Gamete Res..

[24] Ferguson-Smith MA, Yang F, O'Brien PC (1998). Comparative mapping using chromosome sorting and painting. ILAR J..

[25] O’Connor RE (2019). Patterns of microchromosome organization remain highly conserved throughout avian evolution. Chromosoma.

[26] Geurts R, de Jong H (2013). Legume Genomics.

[27] Di Dio C (2020). Analysis of meiotic segregation by triple-color fish on both total and motile sperm fractions in at (1p; 18) river buffalo bull. PLoS One.

[28] Nicodemo D (2009). XY sperm aneuploidy in 2 cattle (*Bos taurus*) breeds as determined by dual color fluorescent in situ hybridization (FISH). Cytogenet. Genome Res..

[29] Pacchierotti F, Adler I-D, Eichenlaub-Ritter U, Mailhes J (2007). Gender effects on the incidence of aneuploidy in mammalian germ cells. Environ. Res..

[30] Pauciullo A (2011). Incidence of XY aneuploidy in sperm of two indigenous cattle breeds by using dual color fluorescent in situ hybridization (FISH). Theriogenology.

[31] Gianaroli L (2010). Predicting aneuploidy in human oocytes: Key factors which affect the meiotic process. Hum. Reprod..

[32] Garcia-Herrero S, Simon B, Garcia-Planells J (2020). The reproductive journey in the genomic era: From preconception to childhood. Genes.

[33] Pawlak P, Pers-Kamczyc E, Renska N, Kubickova S, Lechniak D (2011). Disturbances of nuclear maturation in BCB positive oocytes collected from peri-pubertal gilts. Theriogenology.

[34] Fragouli E, Wells D, Delhanty J (2011). Chromosome abnormalities in the human oocyte. Cytogenet. Genome Res..

[35] Ectors F (1995). Cytogenetic study of bovine oocytes matured in vitro. Theriogenology.

[36] Asakawa T, Ishikawa M, Shimizu T, Dukelow W (1988). The chromosomal normality of in vitro-fertilized rabbit oocytes. Biol. Reprod..

[37] Martin RH (1984). Comparison of chromosomal abnormalities in hamster egg and human sperm pronuclei. Biol. Reprod..

[38] A'arabi S, Roussel J, Chandler J (1997). Chromosomal analysis of mammalian oocytes matured in vitro with various culture systems. Theriogenology.

[39] Munné S (2017). Detailed investigation into the cytogenetic constitution and pregnancy outcome of replacing mosaic blastocysts detected with the use of high-resolution next-generation sequencing. Fertil. Steril..

[40] Mahmoud KGM (2004). Meiotic stages and incidence of diploid oocytes in Egyptian cattle and buffaloes. Assiut Vet. Med. J..

[41] Bertoni A (2020). Similarities and differences between river buffaloes and cattle: Health, physiological, behavioural and productivity aspects. J. Buffalo Sci..

[42] Hassold T, Hall H, Hunt P (2007). The origin of human aneuploidy: Where we have been, where we are going. Hum. Mol. Genet..

[43] Gruhn JR (2019). Chromosome errors in human eggs shape natural fertility over reproductive life span. Science.

[44] Yang C-Y (2021). Molecular signatures of in vitro produced embryos derived from ovum pick up or slaughterhouse oocytes in buffalo. Theriogenology.

[45] Pauciullo A (2014). Development of a sequential multicolor-FISH approach with 13 chromosome-specific painting probes for the rapid identification of river buffalo (*Bubalus bubalis*, 2n= 50) chromosomes. J. Appl. Genet..

[46] Iannuzzi L, Di Berardino D (2008). Tools of the trade: Diagnostics and research in domestic animal cytogenetics. J. Appl. Genet..

