# Supplementary material for: Oocyte aneuploidy rates in river and swamp buffalo types (Bubalus bubalis) determined by Multi-color Fluorescence In Situ Hybridization (M-FISH)
Source: Sci Rep. 2022 May 19;12:8440. doi: 10.1038/s41598-022-12603-9 (PMC9120204; doi:10.1038/s41598-022-12603-9)
Supplement: Supplementary file 1 — Supplementary Figures. [file 41598_2022_12603_MOESM1_ESM.docx]

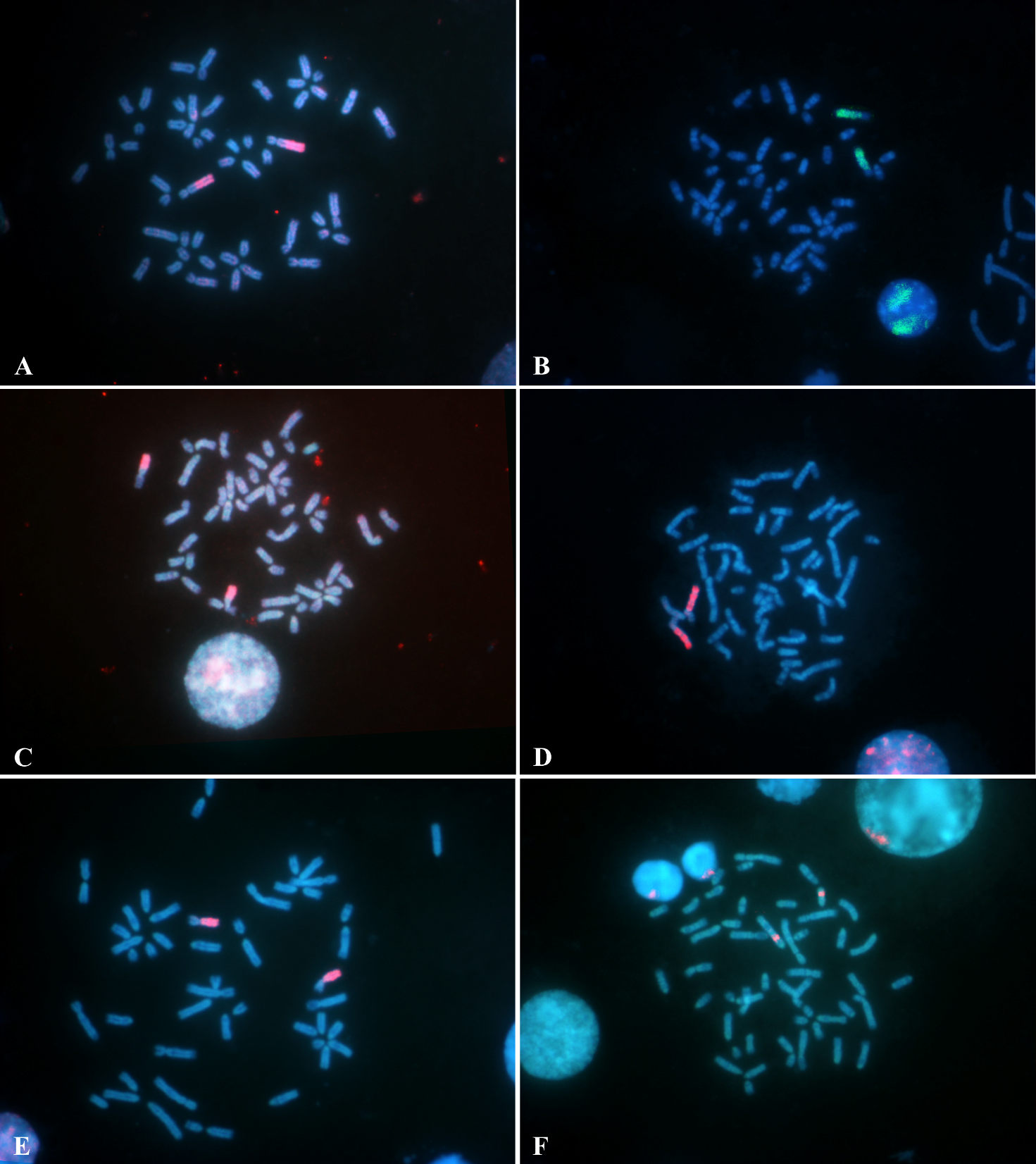


**Supplementary Figure 1.** Specific signals clearly detected on river buffalo (*Bubalus bubalis*, 2n=50) mitosis after FISH with single chromosome painting probes, all labelled in red except for the 2q in green. DAPI was used for the staining. A) 1q; B) 2q; C) 3q; D) 4q; E) 5q; F) X region q21-25 (signal also on the Y-chromosome, region 1.8-1.10).


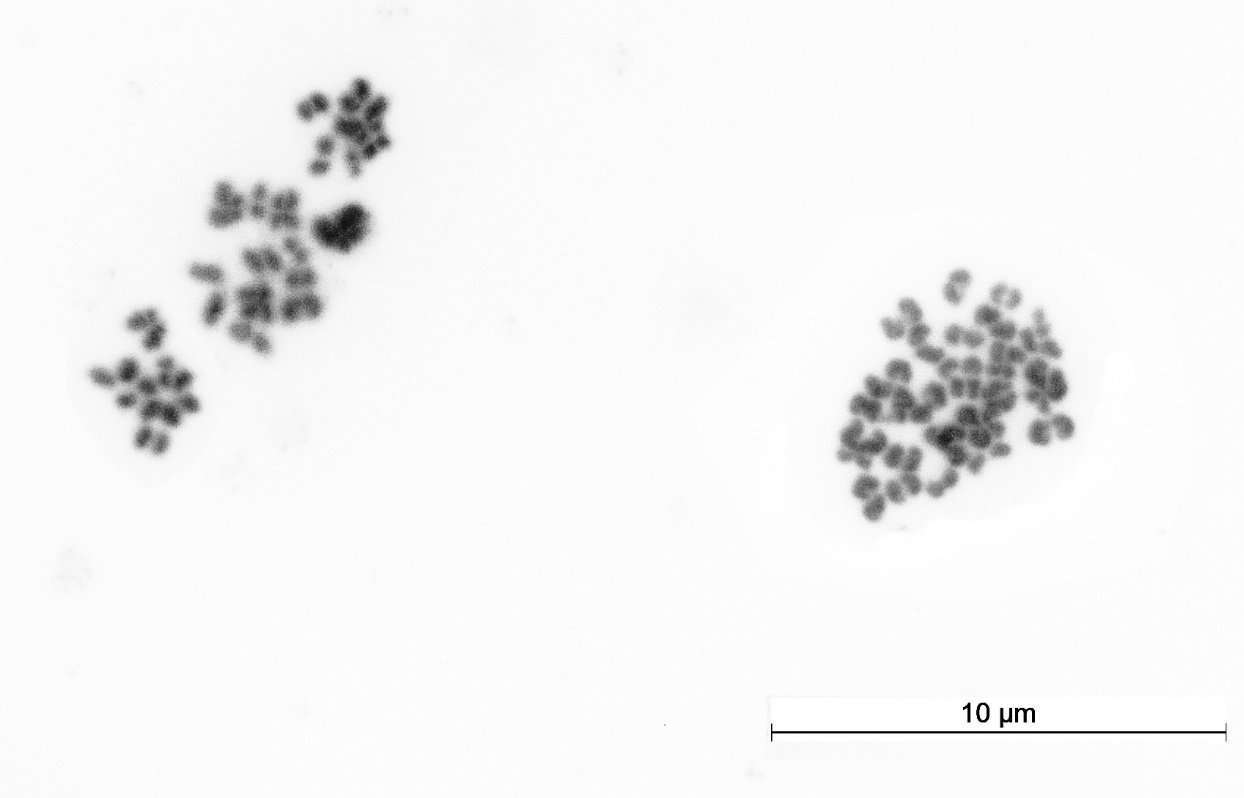


**Supplementary Figure 2.** Metaphase II and corresponding first polar body of *in vitro*-matured secondary oocyte of river buffalo before the sequential multicolour hybridization.
